# Recent Progress in the Systemic Treatment of Advanced/Metastatic Cholangiocarcinoma

**DOI:** 10.3390/cancers12092599

**Published:** 2020-09-11

**Authors:** Raluca Maria Fostea, Elisa Fontana, Gonzalo Torga, Hendrik-Tobias Arkenau

**Affiliations:** 1 Drug Development Unit, Sarah Cannon Research Institute UK, 93 Harley Street, Marylebone, London W1G 6AD, UK; Raluca.Fostea@hcahealthcare.co.uk (R.M.F.); Elisa.Fontana@hcahealthcare.co.uk (E.F.); Gonzalo.Torga@hcahealthcare.co.uk (G.T.); 2 Cancer Institute, University College London, 72 Huntley Street, Bloomsbury, London WC1E 6DD, UK

**Keywords:** cholangiocarcinoma, biliary tract cancers, molecular profiling, driver mutations, targeted therapy, immunotherapy

## Abstract

**Simple Summary:**

The incidence of cholangiocarcinomas is rising. The prognosis of this heterogeneous group of tumors remains poor. Standard chemotherapy options are limited and frequently not effective. Recently, extensive molecular profiling identified key actionable drivers leading to successful biomarker-selected clinical trials. A number of novel targeted therapy drugs and immune checkpoint inhibitors are under investigation. In this review we summarized recent progress in the systemic treatment of cholangiocarcinomas, highlighting biomarkers and therapies in ongoing early-phase clinical trials.

**Abstract:**

Cholangiocarcinomas (CCAs) comprise of a heterogeneous group of cancers arising in the biliary tract (intrahepatic or iCCA, perihilar or pCCA and distal or dCCA; the latter are known under the collective term of eCCA), each subtype having its own particularities in carcinogenesis, management and prognosis. The increasing incidence in recent decades, limited treatment options and high mortality rates, even in the early stages, have led to an imperious need for more in-depth understanding and development of tailored treatments for this type of aggressive tumour. The wide use of molecular profiling has increased the understanding of biology and identified key molecular drivers, for example, *IDH1* mutations or *FGFR2* fusions for iCCA, or *BRAF* mutations in eCCA. Most recently, the FDA approved pemigatinib, an *FGFR* inhibitor and ivosidenib, an *IDH1* inhibitor, but even though progress has been made to better understand the mechanisms of tumorigenesis, genetic make-up, and tumour resistance to standard chemotherapy and targeted therapies, cholangiocarcinomas still represent an important challenge in the daily clinical practice of oncology. The purpose of this review is to highlight the recent progress in the systemic treatment of advanced/metastatic CCAs with a focus on targeted drugs and their biomarkers currently evaluated in early-phase clinical trials.

## 1. Introduction

Cholangiocarcinomas comprise of a heterogeneous group of malignancies arising from the epithelium of the biliary tract, of which approximately 90% are adenocarcinoma. They are anatomically classified as intrahepatic (or iCCA), and extrahepatic (or eCCA) which consist of perihilar or pCCA and distal or dCCA [1].

The highest incidence is seen in Asian countries and parts of South America, where the liver fluke is endemic, whereas Europe, USA or Australasia have a much lower incidence. Recent decades have seen a steady increase in incidence, in particular of iCCA, most likely due to an improvement in diagnostic tools, an increasing number of patients with chronic liver disease (i.e., viral hepatitis or steatohepatitis), dietary intake, migration to western countries, and an increasing number of cholecystectomies; other possible causes, such as environmental toxins, have been described [1].

Surgical treatment options are limited for most patients, as the majority of patients are initially diagnosed with locally advanced or metastatic disease. In this setting, systemic chemotherapy with either a combination of gemcitabine/cisplatin in fit, or gemcitabine monotherapy in frail patients, are standard first line therapy options [2]. Only recently, the ABC-06 study showed that selected patients may benefit from FOLFOX (folinic acid, 5-fluorouracil and oxaliplatin) in the second-line setting, although the benefits were marginal [3]. Moreover, a recent randomised phase-2 study showed that regorafenib compared to the best supportive care improved progression-free survival in patients who received a prior gemcitabine/cisplatin based therapy—the benefit was marginal, with no impact on overall survival [4].

With such modest treatment results [5], and a survival rate of less than 10% at 5 years [6], the need to understand tumour biology and the underlying disease mechanisms is a priority. In this review, recent progress in the systemic treatment of advanced/metastatic cholangiocarcinomas (CCAs) is discussed with a focus on targeted drugs and their biomarkers currently evaluated in early-phase clinical trials.

## 2. Genomic Characterisation by Tumour Site

Historically, the subtypes of CCAs were grouped under the same term of biliary tract cancer and subsequently anatomically classified as iCCA and eCCA (comprising of pCCA and dCCA) often based on their clinical behaviour and outcome.

**Figure 1:**
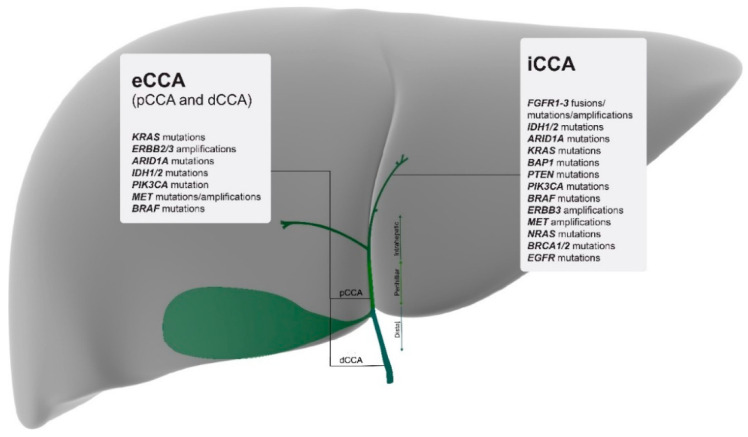
Molecular mapping of the most relevant actionable gene alterations based on tumour location and listed from highest to lowest percentages [12]. iCCA = intrahepatic cholangiocarcinoma; pCCA = perihilar cholangiocarcinoma; dCCA = distal cholangiocarcinoma; eCCA = extrahepatic cholangiocarcinoma.

Only recently, with the advent of larger scale genomic profiling, several studies have defined CCAs by their location and, in addition, have described specific mutations for each anatomical subtype. A comprehensive mapping of the most relevant and targetable mutations for each subtype is listed in Figure 1.

For example, a study conducted by Nakamura et al. on 260 CCAs concluded that iCCAs have the highest levels of *FGFR2* fusions and *IDH1* mutations, whereas eCCAs expressed high levels of *KRAS*, *TP53* and *SMAD4* mutations [7].

Another prospective analysis of 195 patients conducted by Lowery et al., using the MSK-IMPACT platform, has shown that the highest mutated gene for iCCAs was *IDH1* (30%), an AT-rich interaction domain 1A (*ARID1A*—23%), *BAP1* (20%), *TP53* (20%) and *FGFR2* gene fusions (14%). This study also demonstrated that *CDKN2A/B* and *HER2* alterations were associated with a negative prognostic outcome in advanced CCAs [8].

The study conducted by Javle et al. in 554 patients with CCAs demonstrated that *FGFR2* and *IDH1* mutations were most prominent in iCCAs, and that they were mutually exclusive. Their study also concluded that *FGFR2* fusions were associated with better prognosis, while *TP53* and *KRAS* mutations were associated with a poor prognosis—whether patients with *FGFR2*-fusions had a longer overall survival (OS) due to prior treatment with *FGFR* inhibitors remains speculative [9].

Beyond the anatomical location, there is also evidence that molecular alterations are associated with known risk factors, such as liver fluke infection. An analysis by Juskaul et al. in 489 CCAs described four disease clusters, by combining clinical and molecular data. Cluster 1 comprised of mostly fluke-positive tumours; cluster 2 contained a mix of fluke positive and negative tumours. These two clusters significantly presented high percentages in *TP53* mutations and elevated *ERBB2* gene expression. Clusters 3 and 4 comprised mostly fluke-negative tumours: cluster 3 exhibited an upregulation of immune checkpoint genes (*PD-1*, *PD-L2,* and *BTLA*) and pathways related to antigen cross-presentation, CD28 co-stimulation, and T-cell signal transduction; cluster 4 was characterized by *BAP1*, *IDH1/2* mutations, *FGFR* alterations and *PI3K* pathway signatures. The better prognosis was observed in fluke negative tumours harbouring *FGFR2* fusions or *IDH* mutations [10].

Another whole-genome sequencing study on liver fluke-associated CCA, performed by Ong et al. [11] has further discovered, apart from the well-known mutations (*TP53*, *KRAS*), new mutated genes in a small percentage of patients (*MLL3*, *ROBO2*, *RNF34*, *PEG3* and activating mutations of the *GNAS* oncogene), providing a deeper understanding of the genomic landscape in this type of CCA.

## 3. Potential Clinical Applications of Molecular Profiling

A more extensive application of profiling platforms in patients with CCAs led to the increased identification of potentially actionable molecular alterations, with access to biomarker-selected clinical trials. To date, the most promising results have been reported for patients whose tumours harbour *IDH*-mutations, *FGFR2* fusions or *BRAF^V600E^* mutations (Appendix A Table A1). Several other clinical trials using targeted or combination therapies are ongoing (Appendix A Table A2).

### 3.1. FGFR Inhibition

In April 2020, the FDA approved pemigatinib (selective *FGFR1-3* inhibitor) as the first targeted therapy for second-line treatment in locally advanced/metastatic CCAs harbouring *FGFR2* fusions or rearrangements. The FIGHT-202 study, a multicentre, open-label, phase-2 study included 146 patients with *FGFR* alterations—of those, 107 patients had *FGFR2* fusions or rearrangements, 20 patients had other *FGF/FGFR* alterations, 18 patients had no *FGF/FGFR* alterations, and one patient had an undetermined *FGF/FGFR* alteration. After a median follow-up of 17.8 months, the overall response rate (ORR) was 36% (38 patients with *FGFR2* fusions or rearrangements), out of which three patients had a complete response (CR) and 35 had partial responses (PR). Of the 38 patients who had a response, 24 patients (63%) had a response lasting at least 6 months, and seven patients (18%) had a response lasting at least 12 months. Generally, the treatment was well tolerated, with some *FGFR*-specific side effects including hyperphosphatemia, stomatitis, and nail changes—grade 3 or worse adverse events (AE) were hypophosphatemia (18 patients), arthralgia (nine patients), stomatitis (eight patients), hyponatremia (eight patients), abdominal pain (seven patients) and fatigue (seven patients). Seventy-one cancer-related deaths were reported, and no deaths were treatment-related [13,14].

In another phase-2 study in patients with chemotherapy-refractory *FGFR*-altered CCAs, the *FGFR1-3* inhibitor infigratinib showed clinical activity in patients with *FGFR2*-fusions. In the sixty-one patients who enrolled into this study (48 patients with *FGFR2* fusion, eight patients with *FGFR* mutation, and three patients with *FGFR* amplification) the ORR was 14.8% (*FGFR2* fusion only), and median progression-free survival was 5.8 months. Again, *FGFR*-specific side effects consisted of hyperphosphatemia (72.1%), fatigue (36.1%), stomatitis (29.5%) and alopecia (26.2%) [15].

Although response rates and progression-free survival (PFS) of these early studies are clinically relevant and promising, some of the responses have been short lived and further investigation into resistance mechanisms have been made. In this context, a recent study showed that acquired resistance to the *FGFR* inhibitor infigratinib (BGJ1398) is caused by point-mutations in *FGFR2*, with the most prominent being the gatekeeper mutation *p.V564F*, which was only present in the post-progression analyses in plasma and new metastatic sites. Apart from the new *FGFR2* point mutations, alterations in the *PTEN/PI3K* pathway were also discovered as a potential mechanisms of resistance [16].

Another phase-1/2 study of the irreversible pan-FGFR inhibitor (FGFR 1-4), TAS-120, futibatenib, enrolled 45 chemotherapy-refractory CCA patients harbouring FGFR aberrations (FGFR2 fusions, mutations, amplifications or re-arrangements) and resulted in an ORR of 25% and a disease control rate (DCR) of 78.6%. Interestingly, 13 patients received prior treatment with at least one reversible FGFR-inhibitor, and of those, four patients achieved a partial response [17,18].

Importantly, these findings were also confirmed in a small study of futibatenib in patients with acquired resistance to prior *FGFR* inhibition. Of the six patients enrolled into this study, two patients achieved a PR, and two patients had stable disease, with a durable response benefit between 5.1 to 17.2 months [19].

### 3.2. IDH1 Inhibition

Another recent breakthrough has been reported in CCA patients whose tumours harboured isocitrate dehydrogenase 1 (*IDH1*) mutations. The randomized, double-blinded phase-3 study (ClarIDHy study) investigated the role of the *IDH* inhibitor, ivosidenib, versus placebo in patients with advanced CCAs. This study showed for the first time a significant PFS benefit for patients who received ivosidenib—the 6 and 12 months PFS rates were 32.0% and 21.9% for ivosidenib versus 0% in the placebo arm. Although the ORR was marginal at 2.4%, 50.8% of patients achieved stable disease, amounting to a 52% disease control rate. The median OS in the ivosidenib arm was 10.8 months compared to 9.7 months in the placebo arm—importantly, this included 57% patients who crossed-over from the placebo to the ivosidenib arm. The treatment was well tolerated, and the most common treatment-related adverse events were nausea (32.1%), diarrhoea (28.8%), fatigue (23.7%), cough (19.2%), abdominal pain (18.6%), ascites (18.6%), decreased appetite (17.3%), anaemia (16%) and vomiting (16%) [20].

### 3.3. BRAF Inhibition

The inhibition of the *BRAF^V600E^* gene mutation through *BRAF* and *MEK*-inhibitors is an established treatment in a variety of cancers including melanoma, thyroid cancer and non-small cell lung cancer (NSCLC).

In this context, the phase-2 basket study, ROAR, evaluated the combination of dabrafenib (*BRAF* inhibitor) and trametinib (*MEK*-inhibitor) in 33 patients with CCA. The ORR was 41%, with six out of 13 responses ongoing at the data cut-off and the duration of response (DOR) ≥ 6 months was 54%. The median PFS and OS was 7.2 months and 11.3 months, respectively. The treatment resulted in a typical adverse event profile seen in other cancers and predominantly included nausea, fatigue, skin rashes, fever and diarrhoea [21].

In addition, several case series have published good tolerability and response to this combination [22,23,24] and another phase-2 trial, VE-BASKET, demonstrated single agent efficacy of the *BRAF* inhibitor, vemurafenib, in *BRAF^V600E^* mutant cancers. In this study, a total of 208 patients with 26 different types of cancers were enrolled, including nine CCA patients. Three patients obtained a partial response, four had stable disease and two patients progressed on trial treatment. The duration of treatment was sixty-one days for the cholangiocarcinoma arm [25].

### 3.4. HER-2 Inhibition

Alterations, in particular *HER*-amplifications, in the *HER-2* receptor family have been shown to drive some of the tumour signalling in patients with CCA. Although results of early phase-2 studies of lapatinib [26] or erlotinib + sorafenib [27] did show disappointing results, several studies of *HER-2*-directed therapies alone or in combination with chemotherapy are currently evaluated. In particular, the results of the BILHER phase-2 study (trastuzumab in combination with gemcitabine plus cisplatin) are awaiting (NCT03613168).

Early results of the phase-2 basket study, MyPathway, evaluated the efficacy and safety of targeted therapies in non-approved tumour types which harboured relevant genetic alterations. Of note, the combination of the *HER-2*-directed antibodies, trastuzumab and pertuzumab, demonstrated promising results in the 11 *HER-2* (amplification/overexpression) positive CCA patients. Four patients achieved a PR and another three patients had stable disease (SD), with a PFS ranging between 2.8 and 4.2 months—final results of this study are awaiting [28].

Other HER-2-directed therapies, such as the antibody drug conjugate (trastuzumab-emtansine, TDM-1), have demonstrated promising results in preclinical studies [29] and clinical trials in CCA are planned.

### 3.5. Anti-Angiogenesis Targeting

The REACHIN clinical trial was a randomized phase-2 study evaluating the safety and efficacy of regorafenib in patients with unresectable/metastatic CCA who progressed after gemcitabine/platinum chemotherapy. Although no radiological response was seen, 74% of patients had disease stabilization amounting to a median PFS of 3 months compared to 1.5 months in the placebo arm. The modest improvement in PFS did not result in an improved median OS, which was 5.3 and 5.1 months, respectively [4].

Another recent phase-1 study explored the role of the dual inhibition of the *VEGFR* and *PD-1* pathways, using a combination of ramucirumab and pembrolizumab in 26 patients with pre-treated advanced or metastatic CCAs. The trial has shown limited clinical benefit with only one patient achieving a partial response and another nine patients had stable disease. In patients whose tumours were *PD-L1* positive (*n* = 12), the median PFS was 1.5 months with an 11.3 months median OS, compared to patients with *PD-L1* negative tumours (*n* = 12), whose median PFS was 1.6 months with a median of OS 6.1 months, respectively. The authors concluded that this combination was not worth exploring further [30].

### 3.6. DDR Targeting

Other potentially targetable molecular alterations in CCAs are germline and somatic mutations in the *BRCA 1 and 2* genes. Specifically, a recent multicentre retrospective analysis conducted between 2000 and 2013 of five participating institutions in Israel and the USA, identified 18 patients diagnosed with CCA to have either a germline or somatic *BRCA1/2* mutation. In this analysis, four patients received a *PARP* inhibitor in the advanced setting after the failure of standard chemotherapy, with one patient achieving a PFS of 43 months and an OS of 65 months and the other three patients had a PFS of 3.7, 2.0 and 4.7 months, with an OS of 33, 11 and 32.8 months respectively [31].

A variety of *PARP* inhibitors are currently used in clinical trials to investigate their role not only in *BRCA* mutant tumours, but also in gene alterations of the DNA Damaging Repair pathway (*DDR*).

### 3.7. Other Targeted Therapies

With the wide use of multi-gene panel testing, rare genetic alterations such as *RET* or tropomyosin receptor kinase (*TRK*) fusions are increasingly identified in a variety of tumour types including CCA. For example, the *RET*-inhibitor, pralsetinib, and the *TRK* inhibitors, entrectinib and larotrectinib, have recently been approved in a tumour agnostic approach, meaning that once a *RET* or *NTRK*-fusion has been identified, treatment with a *RET* or *TRK* inhibitor can be instigated regardless of tumour origin [32]. Although the total numbers of enrolled CCA patients have been low in the ARROW phase-1 trial, the response rate and duration of response have been encouraging—for example, one patient with heavily pre-treated CCA had a partial response which lasted for 7.5 months [33].

Even though significant progress has been made through modern molecular profiling platforms, contributing to a better understanding of CCA, most results from clinical trials using targeted therapies have been short lived, highlighting the heterogeneity of these tumours and the necessity to advance our understanding of tumour biology and secondary resistance, and to improve the outcomes of treatment by using a combination of targeted therapies [34].

## 4. Immunotherapy Approaches

### 4.1. Immunology of CCAs

The tumour microenvironment and the role of the immune system in the mechanisms around tumours arising in the biliary tree have been studied in depth, highlighting the link between the innate immune system of the liver, the role of T-lymphocytes infiltration and the immune tolerance specific to liver and biliary tract cancers.

Briefly, the hepatic immune environment consists of the highest percentage of the total body macrophage population (between 80% and 90%), also known as Kupffer cells, alongside natural killer (NK) cells. Kupffer cells have a role in inducing immunological tolerance in the liver, a key mechanism exploited by tumour cells to promote tumorigenesis and progression through immune tolerance [35].

Goeppert et al. [36] analysed the impact of tumour-infiltrating immune cells on CCA outcomes, suggesting that intraepithelial infiltrating CD4+ and CD8+ T lymphocytes resulted in better overall survival, which was also seen in patients having a higher total regulatory T lymphocytes; the conclusion of this study was that the adaptive immune system plays a role in fighting tumour cells, potentially leading to longer survival.

Another extensive immunohistochemistry study of 652 tumours described a correlation between *PD-1/PD-L1* expression and genomic mutations [37]. Although *PD-L1* expression was found in only a small percentage (8.6%), with the highest presence in GBC (12.3%), followed by iCCA (7.3%) and eCCA (5.2%), this was significantly correlated with the presence of *TP53*, *BRAF*, *BRCA2*, and *RNF43* mutations.

Other clinically relevant/potentially targetable mutations found in the *PD-L1* positive samples were: *ARID1A* (50%), *ATM* (3.7%), *BAP1* (3.6%), *BRCA1* (1.9%), *CDKN2A* (11.8%), *ERBB2* (1.8%), *IDH1* (1.8%), *IDH2* (1.8%), *JAK1* (1.9%), *KRAS* (23.2%), *MAP2K* (3.6%), *MSH2* (1.9%), *NRAS* (5.4%), and *PIK3CA* (3.8%). Other relevant biomarkers tested were high tumour mutational burden (TMB, median >17 mutations per megabase) which was found in 10.7% of tumours, and microsatellite instability-high (MSI-H) in 7.1%. *FGFR2* fusions were also identified, but only in the PD-L1 negative cohorts.

A retrospective analysis including 320 CCA patients demonstrated a significant correlation between high *PD-1/PD-L1* expression and advanced, unresectable iCCAs, hepatitis B virus (HBV) infection and poor prognosis. Conversely, in patients with cholelitiasis, *PD-1/PD-L1* levels were downregulated. These findings suggest that the *PD-1/PD-L1* pathway may play an important role in carcinogenesis and the progression of HBV-positive iCCAs [38].

The established role of the immune system and hepatic environment, including the role of chronic inflammation in carcinogenesis, supported the investigation of immune checkpoint inhibitors in cholangiocarcinoma.

### 4.2. Checkpoint Inhibitors

In June 2020, the *PD-1* inhibitor, pembrolizumab, was approved by the FDA for MSI-H/dMMR and TMB-high non-colorectal cancers (TMB >10 per megabase), after the encouraging results of the KENOTE-158 study. Briefly, this phase-2 study enrolled 233 non-colorectal, MSI-H/dMMR patients, out of which 22 were patients with CCAs. All patients enrolled did have disease progression after at least one line of systemic therapy in the advanced/metastatic setting. The ORR was 40.9%, with a median PFS of 4.2 months and a median OS of 24.3 months [39].

In patients with dMMR non-colorectal cancers, the phase-2 NCI-MATCH study enrolled three patients with CCA—of those, one patient had a complete response and two patients had disease progression. For the entire cohort, the ORR was 36%, and the CRR was of 7% with a median PFS of 6.3 months and OS of 17.3 months, respectively [40]. These data are supported by a larger dMMR cohort of 86 patients who received pembrolizumab which also included four CCA patients. For the entire cohort, the ORR was 53%, out of which 21% achieved a complete response. Of the CCA patients enrolled, one patient achieved complete response while the other three patients had stable disease [41].

A combinatorial approach of the *PD-L1* inhibitor, atezolizumab, and the small molecule *MEK*-inhibitor, cobimetinib, was studied in a randomized phase-2 study of 77 patients with *PD-L1* positive CCA. Although response rates were marginal, the disease was controlled in 45.2% of patients in the combination arm, and 32.4% in the monotherapy arm. The trial met its primary endpoint, with a median PFS of 3.65 months in the combination cohort versus 1.87 months in the atezolizumab monotherapy cohort—at this point, mature OS data are awaited [42].

In an unselected biomarker, a single agent checkpoint inhibitor phase-2 study of nivolumab in 54 CCA patients, 10 patients had partial response, and 17 stable disease amounting to a disease control rate of 59%, a median PFS of 3.9 months and OS 14.2 months, respectively. A retrospective analysis showed that all patients who responded had MMR proficient tumours, and out of the entire cohort, 18 patients had *PD-L1* positive tumours; of note 9 out of 10 responding patients had *PD-L1* positive tumours [43].

Currently, there are several phase-2 studies ongoing that are exploring the role of immunotherapies in patients with CCA. Critically, the selection of the appropriate biomarker, understanding tumour biology and the microenvironment and the choice of the right combination will guide us as to whether immunotherapy beyond dMMR and high TMB will have a future in CCA.

## 5. Conclusions

In recent years, significant progress has been made in understanding the biology of CCAs, subsequently leading to improved outcomes for a subgroup of patients with known genetic alterations. For example, the recent success of trials targeting *FGFR2* fusions (FIGHT202 trial) or *IDH1* mutations (ClarIDHy trial) have led to recent approvals of new drugs such as pemigatinib (*FGFR2* inhibitor) and ivosidenib (*IDH1* inhibitor) as efficient subsequent treatment options for patients failing the first line of systemic therapies. Encouraging results have also been obtained by targeting rare genetic mutations like *TRK*- and *RET* fusions in tumour agnostic clinical trials.

The approval of the *PD-1* inhibitor, pembrolizumab, for MSI-H, dMMR and TMB-high tumours has also led to significant survival benefits for patients who fall into this category, as shown in the KEYNOTE-158 trial [39].

Although the therapeutic options for CCA has shifted towards a more targeted approach, leaving behind the “one size fits all” chemotherapy regimens, the challenge still remains for a percentage of patients who do not harbour relevant targetable molecular alterations.

Future research in patients with CCA has to be the understanding of primary and secondary resistance, as many of the targeted therapies have short-lived responses. In this context, biomarker-driven clinical trials have to guide us towards the best combinatorial approach of new drugs. In addition, a deeper understanding of the tumour and host immune environment and subsequently the selection of the right immune response biomarkers has to be studied.

## Appendix A

**Table A1:** Completed clinical trials using targeted/combination therapies in patients with CCAs. NA: not available.

Drug (*Mechanism of Action*)	Target	Phase	Total Number of CCA Patients Enrolled	Outcome in CCA
Pemigatinib (*FGFR1-3 inhibitor*)	FGFR alterations	2	146	ORR = 36% CR (*n* = 3) PR (*n* = 35) DOR > 12 mo (*n* = 7) [13,14]
Infigratinib (*FGFR1-3 inhibitor*)	*FGFR2* fusions	2	61	ORR = 14.8% PFS = 5.8 mo [15]
Futibatenib (*FGFR 1-4 inhibitor*)	FGFR2 fusions, mutations, amplifications or re-arrangements	1/2	45	ORR = 25% PR (*n* = 4) [17,18]
Futibatenib (*FGFR 1-4 inhibitor*)	FGFR alterations + acquired resistance to FGFR inhibitors	N/A	6	PR (*n* = 2) SD (*n* = 2) [19]
Ivosidenib (*IDH1 inhbitor*)	*IDH1* mutations	3	185	ORR = 2.4% PFS = 32% at 6 mo; 21.9% at 12 mo; OS = 9.7 mo [20]
Dabrafenib (*BRAF^V600E^ inhibitor*) Trametinib *(MEK-inhibitor)*	*Ras-Raf*-MEK-*ERK pathway* *BRAF^V600E^* mutation	2 (basket)	33	CCA cohort: ORR = 41% PFS = 7.2 mo OS = 11.3 mo [21]
Vemurafenib (*BRAF^V600E^ inhibitor*)	*BRAF^V600E^* mutation	2 (basket)	9	PR (*n* = 3) SD (*n* = 4) CCA survival data NA [25]
Trastuzumab Pertuzumab (anti-HER2-antibodies)	*HER2* (amplification/overexpression)	2 (basket)	11	PR (*n* = 4) SD (*n* = 3) PFS 2.8 mo–4.2 mo [28]
Regorafenib (*tyrosine kinase inhibitor*)	Angiogenesis	2	66	PFS 3.0 mo vs. 1.5 mo, HR 0.49 p: 0.004 [30]
Ramucirumab (*anti-VEGFR*) Pembrolizumab *(anti- PD-1)*	Angiogenesis Immune checkpoint	1	26	ORR = 4% PFS = 1.6 mo OS = 6.4 mo [31]
Pralsetinib (*RET inhibitor*)	*RET* mutation	1 (basket)	2	PR (*n* = 1) DOR > 7.5 mo CCA survival NA [34]

**Table A2:** Ongoing (active/recruiting) clinical trials using targeted/combination therapies in patients with cholangiocarcinomas (CCAs).

Drug	Target	Phase	Trial Number
Derazantinib	*FGFR2* fusion	II	NCT03230318
BGJ398 (infigratinib)	*FGFR2* mutation	II	NCT02150967
BGJ398 + Gemcitabine + Cisplatin	*FGFR2* fusion/translocation	III	NCT03773302
Oral Infigratinib	*FGFR 1-3* fusion or other *FGFR* alterations	II	NCT04233567
TAS-120	*FGFR2* fusion, mutation, rearrangement or amplification	I/II	NCT02052778
INCB062079	*FGF/FGFR* alterations	I	NCT03144661
FT 2102 Nivolumab Gemcitabin+Cisplatin	*IDH1* mutation	IB/II	NCT03684811
Olaparib	*IDH1*/2 mutation	II	NCT03212274
AG-120	*IDH1* mutation	III	NCT02989857
Olaparib and ceralasertib	*IDH1/2*-mutation	II	NCT03878095
IDH305	*IDH* mutations	I	NCT02381886
ABM-1310	*BRAF* mutation	I	NCT04190628
Lenvatinib plus Pembrolizumab	*VEGFR*, *FGFR*, *PDGFR*α, *RET*, *KIT*PD-1 checkpoint	II	NCT03797326
Niraparib	*BAP1* and other DNA Damage Response (DDR) Pathway alterations	II	NCT03207347
Rucaparib plus Nivolumab	DNA Damage Response (DDR) Pathway alterations PD-1 checkpoint	II	NCT03639935
GEMOX Cetuximab Trastuzumab Gefitinib Lapatinib Everolimus Sorafenib Crizotinib	Mutations or abnormal activation of HER-2 receptor tyrosine kinase signalling pathway	II	NCT02836847
Pevonedistat Paclitaxel+Carboplatin	*NEDD8*-activating enzyme	II	NCT04175912
ABC294640 (Opaganib)	Sphingosine-kinase2	I/II	NCT03377179
Aldesleukin Autologous CD8+ T Cell Therapy Pembrolizumab Cyclophosphamide	Targeted therapy CD8+ T Cell therapy	I	NCT02757391
A166	*HER-2* Antigen or Amplified *HER-2* Gene	I/II	NCT03602079
Nedisertib, Avelumab and hypofractionated radiation	DNA-dependent protein kinase	I/II	NCT04068194
Atezolizumab Cobimetinib	Immune checkpoint inhibitor *MEK*	II	NCT03201458
Nivolumab Ipilimumab	*CTLA-4 PD-1* checkpoints	II	NCT02834013
Guadecitabine Durvalumab	DNA methyltransferase and immune checkpoint	I	NCT03257761
